# Poisoning by Cantharides
*Midland Reporter, No. XI.


**Published:** 1831

**Authors:** 


### POISONING BY CANTHARIDES.*

A young woman serving in a dram shop administered some lytta in raspberry brandy to two young men, each of them taking half a pint of the liquor in question. One of them soon afterwards set out for Stourport, six miles distant, but was seized on the road with violent pain in the stomach and bowels, which compelled him to sit down frequently, insomuch that he was six hours in reaching the place in question. Mr. Williams of Bewdley was summoned, and found him suffering from violent pain in the stomach, bowels, kidneys, and bladder; a constant desire to make water; and a burning heat in the throat. Vomiting was produced by a mixture of sugar, water, and sweet oil, and afterwards he was ordered to drink freely of linseed tea.

At 4, a. m. next morning the skin was very hot, the sense of burning in the throat more urgent, the straining to make water incessant, whilst only a few drops of blood were voided, the pulse 100, the tongue thickly coated. *V. S. ad* ℥xij.—*Enema, ol. ric.* ℥iss. At noon the strangury continued, with much tenderness in the region of the kidneys, ureters, and bladder; bowels opened; pulse 100. *V. S. ad* ℥xiv.—*Rep. ol. ric. et enemata. Linseed tea and barley water.* At 5, p. m. the tenderness was much increased; pulse 115; great restlessness, with occasional delirium. *V. S. ad* ℥xvj.—*Ol. ric. et enemata.* 12, p. m. Restlessness very great; constant delirium with some slight convulsion; face much flushed and conjunctivæ injected; strangury distressing; pulse 125, hard and incompressible. He was bled to ℥xxiv., kept in a state of syncope for nearly an hour, then allowed to lie down, and eight drops of laudanum in some linseed tea were given him. After this the urgent symptoms ceased, though the urine continued somewhat bloody, and

* Midland Reporter, No. XI.

the tongue peculiarly coated till the 6th or 7th of July. The patient was then convalescent.

We need not give the particulars of the other case, as the symptoms were similar to those of the last, and the same kind of treatment, though not so active, proved successful.

